# Supplementary figures and images for: Fracture healing is delayed in the absence of gasdermin-interleukin-1 signaling
Source: eLife. 2022 Mar 4;11:e75753. doi: 10.7554/eLife.75753 (PMC8923664; doi:10.7554/eLife.75753)

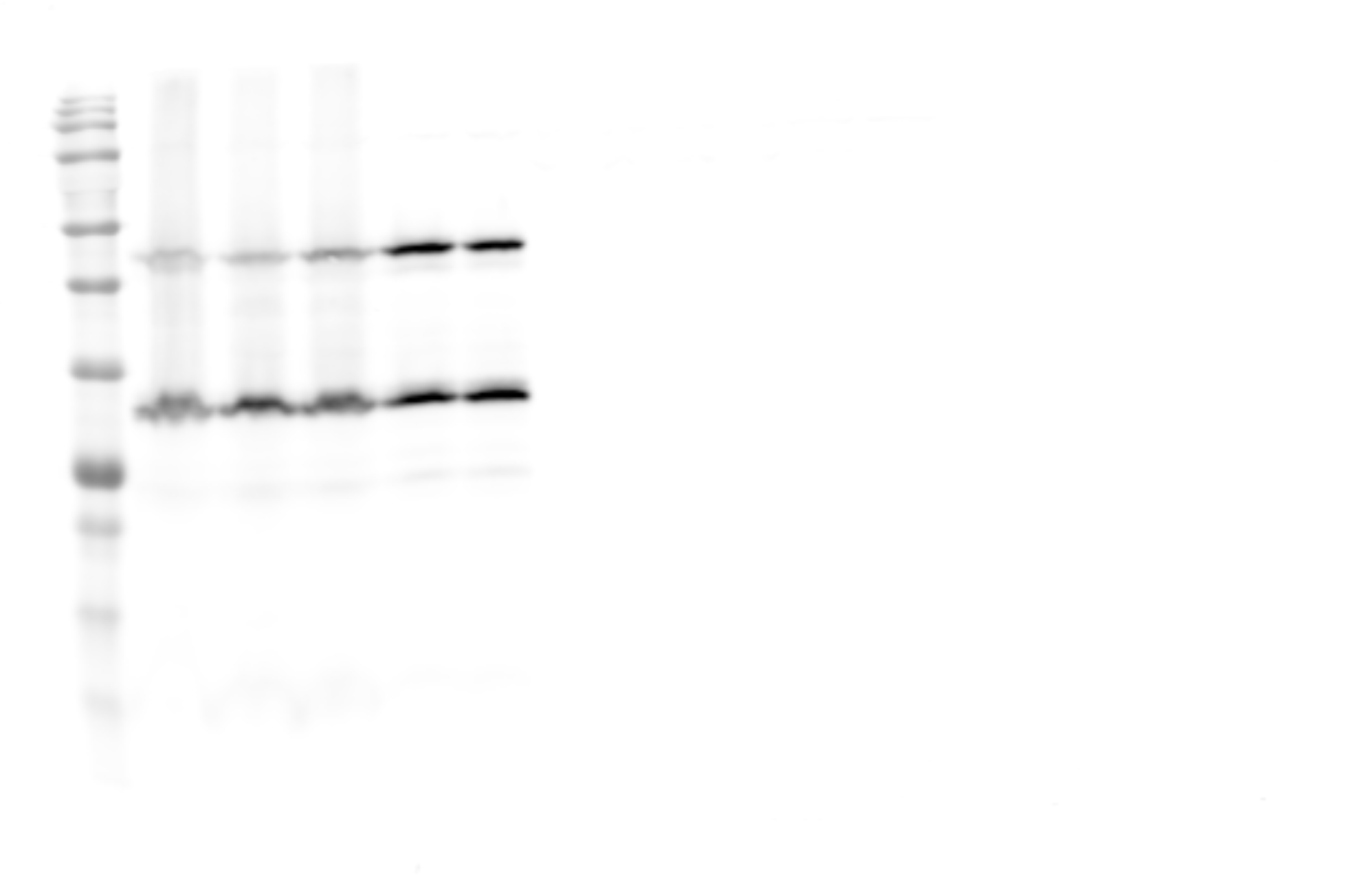

Supplement: Figure 1—source data 2. [file elife-75753-fig1-data2.zip › source data Figure 1C and D/Fig. 1c_gsdmd.tif]

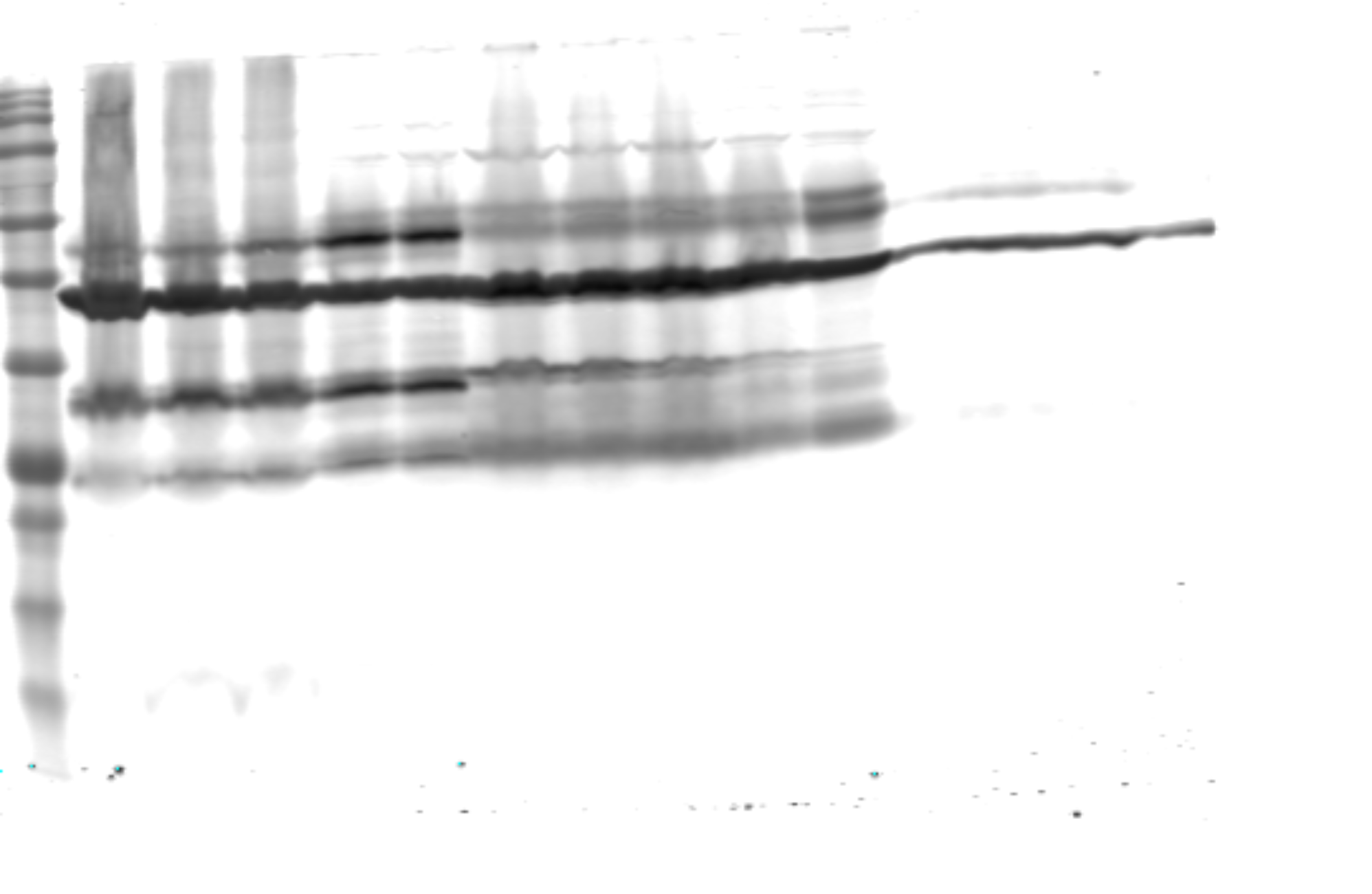

Supplement: Figure 1—source data 2. [file elife-75753-fig1-data2.zip › source data Figure 1C and D/Fig. 1c_actin.tif]

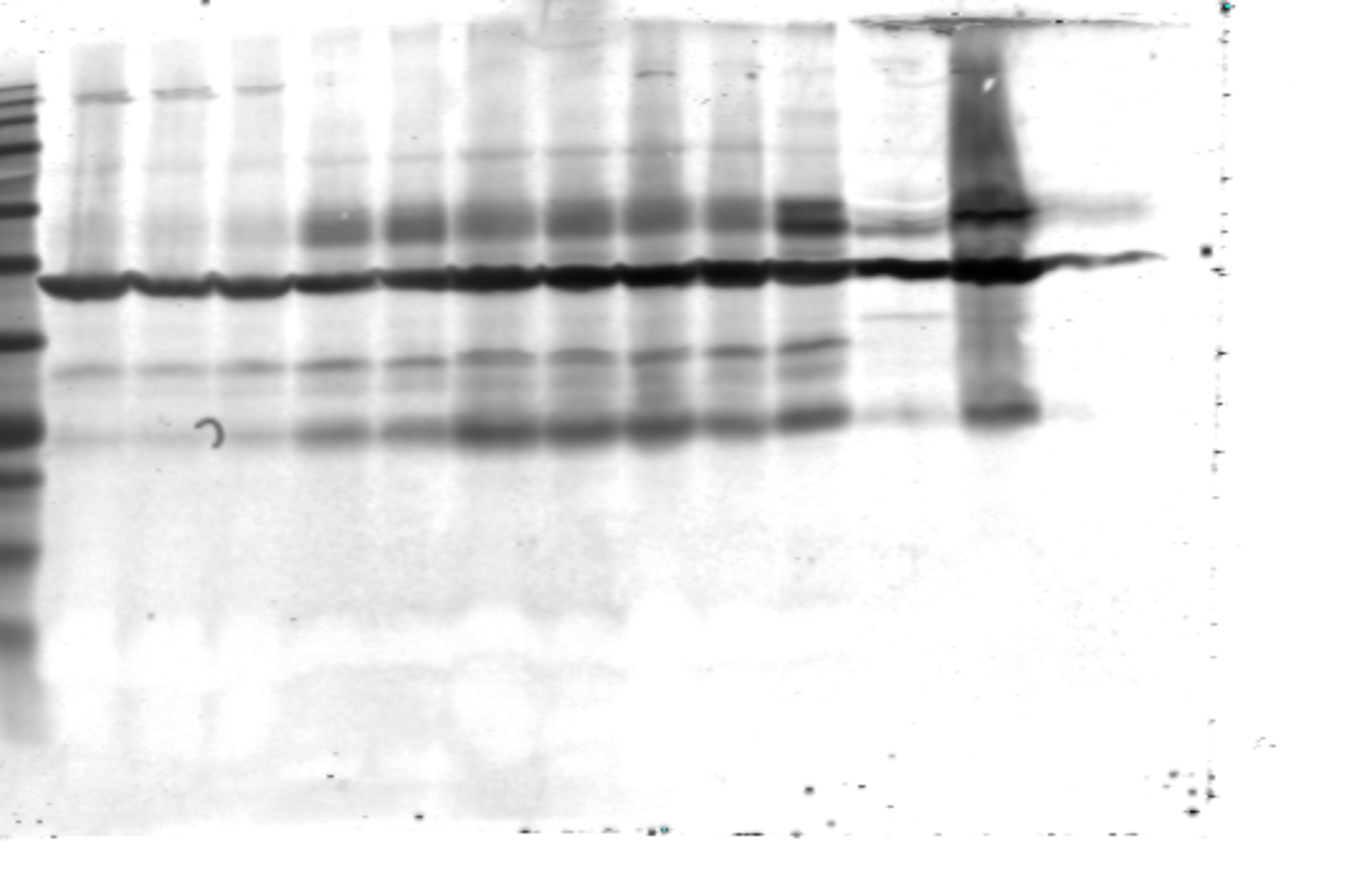

Supplement: Figure 1—source data 2. [file elife-75753-fig1-data2.zip › source data Figure 1C and D/Fig. 1d_actin.tif]

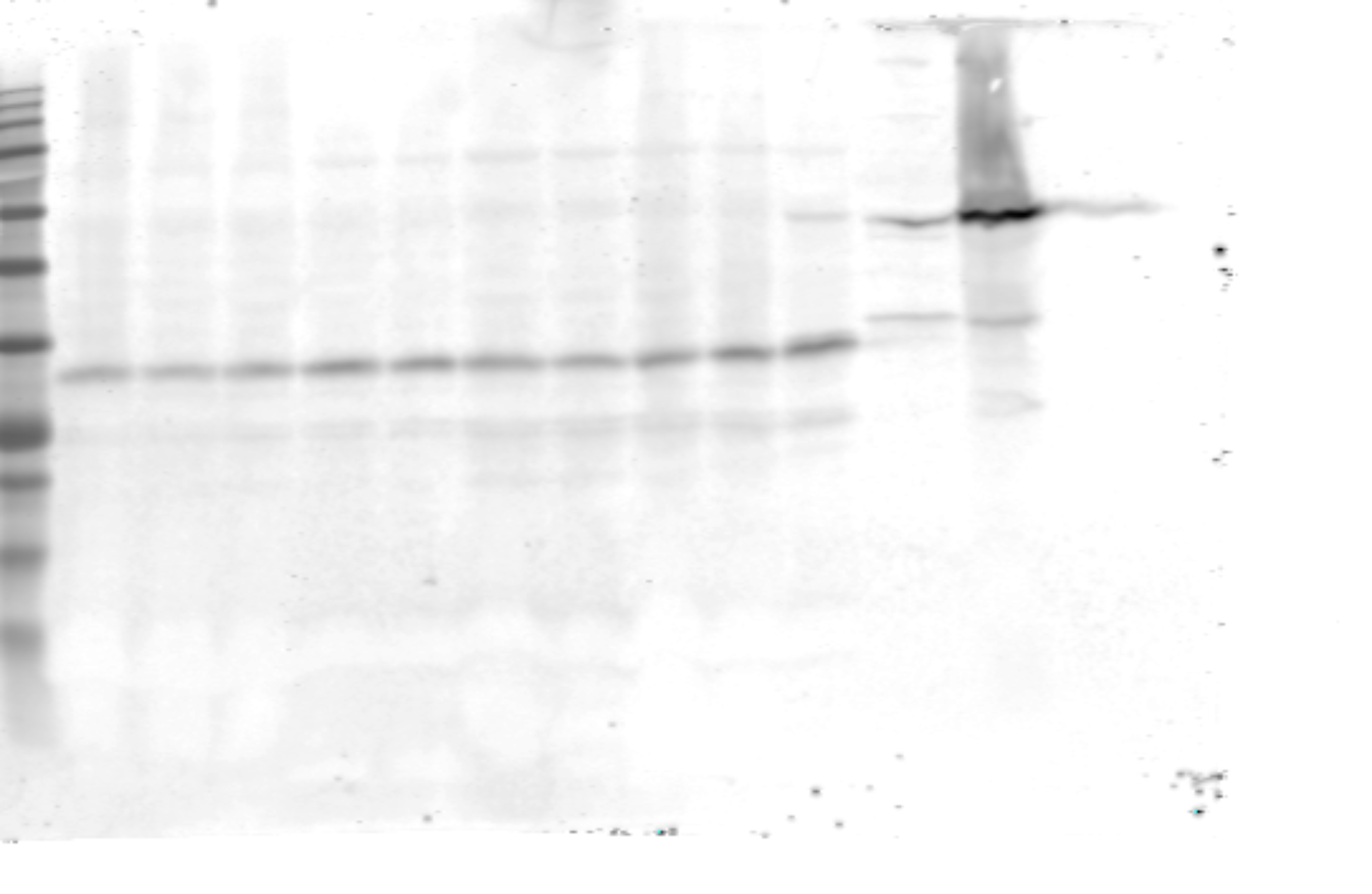

Supplement: Figure 1—source data 2. [file elife-75753-fig1-data2.zip › source data Figure 1C and D/Fig. 1d_gsdme.tif]

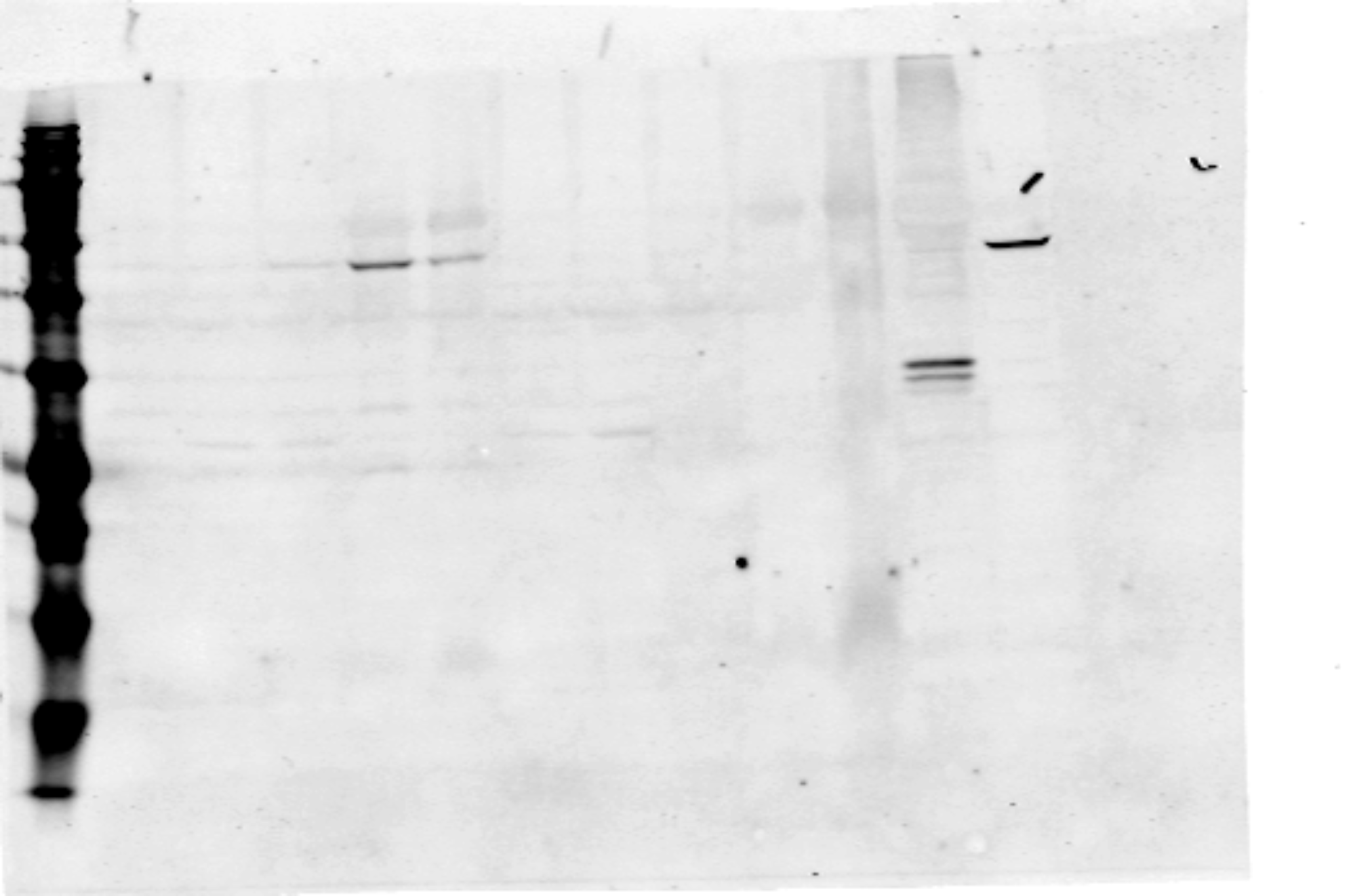

Supplement: Figure 1—figure supplement 2—source data 2. [file elife-75753-fig1-figsupp2-data2.zip › source data Figure 1-figure supplement 2A and B/Fig. S2a_GSDMD.tif]

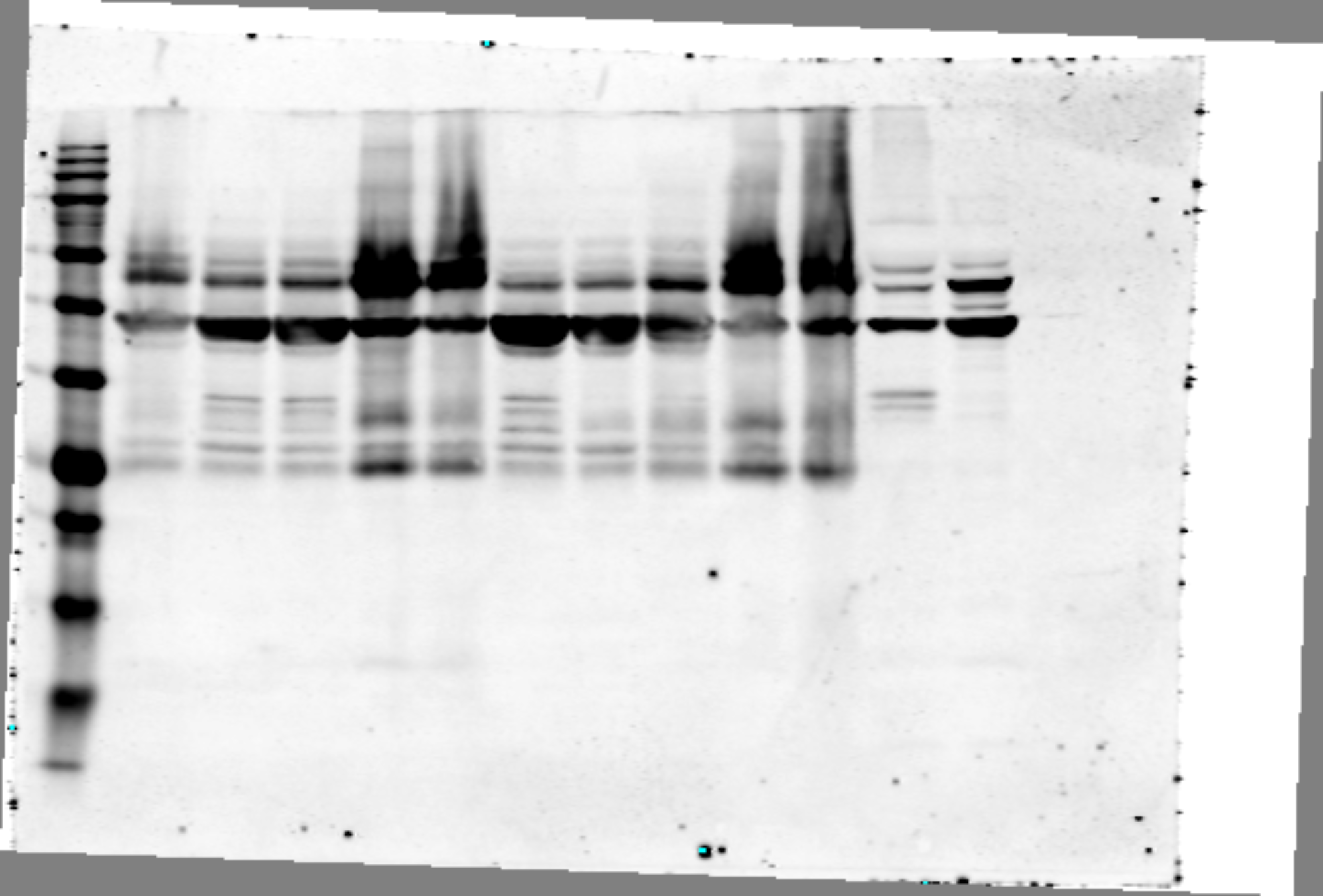

Supplement: Figure 1—figure supplement 2—source data 2. [file elife-75753-fig1-figsupp2-data2.zip › source data Figure 1-figure supplement 2A and B/Fig. S2a_actin.tif]

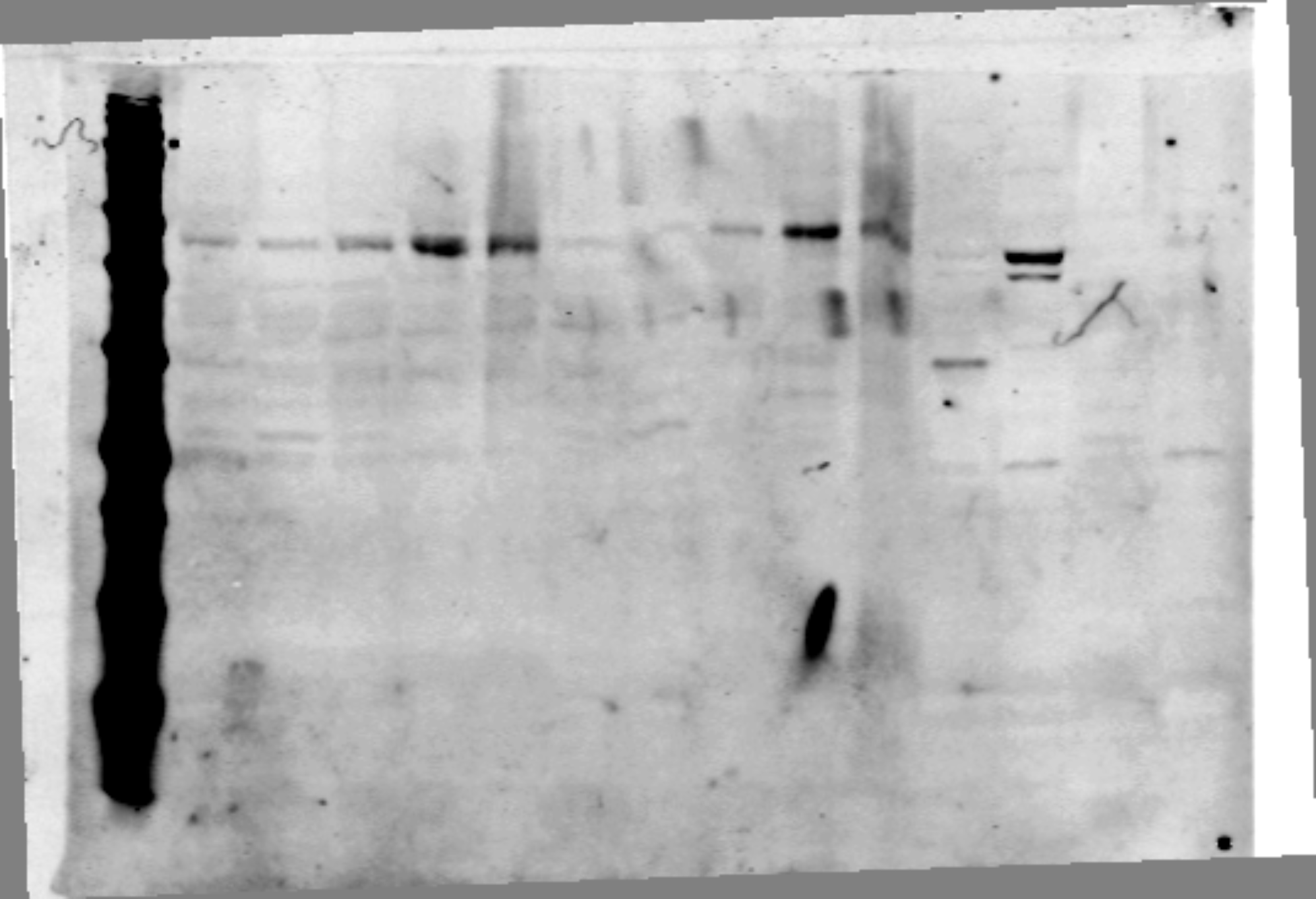

Supplement: Figure 1—figure supplement 2—source data 2. [file elife-75753-fig1-figsupp2-data2.zip › source data Figure 1-figure supplement 2A and B/Fig. S2b_GSDME.tif]

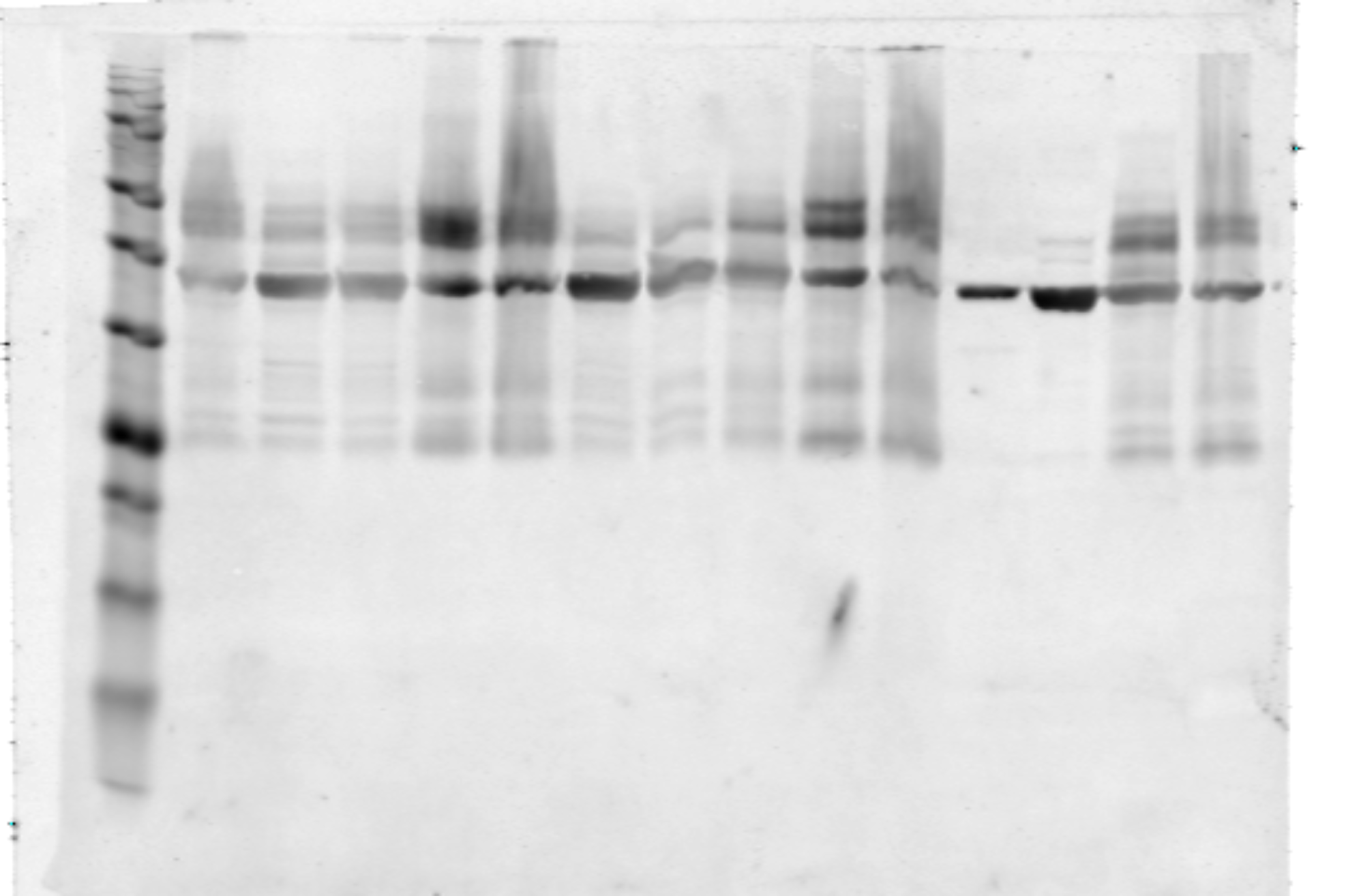

Supplement: Figure 1—figure supplement 2—source data 2. [file elife-75753-fig1-figsupp2-data2.zip › source data Figure 1-figure supplement 2A and B/Fig. S2b_actin.tif]

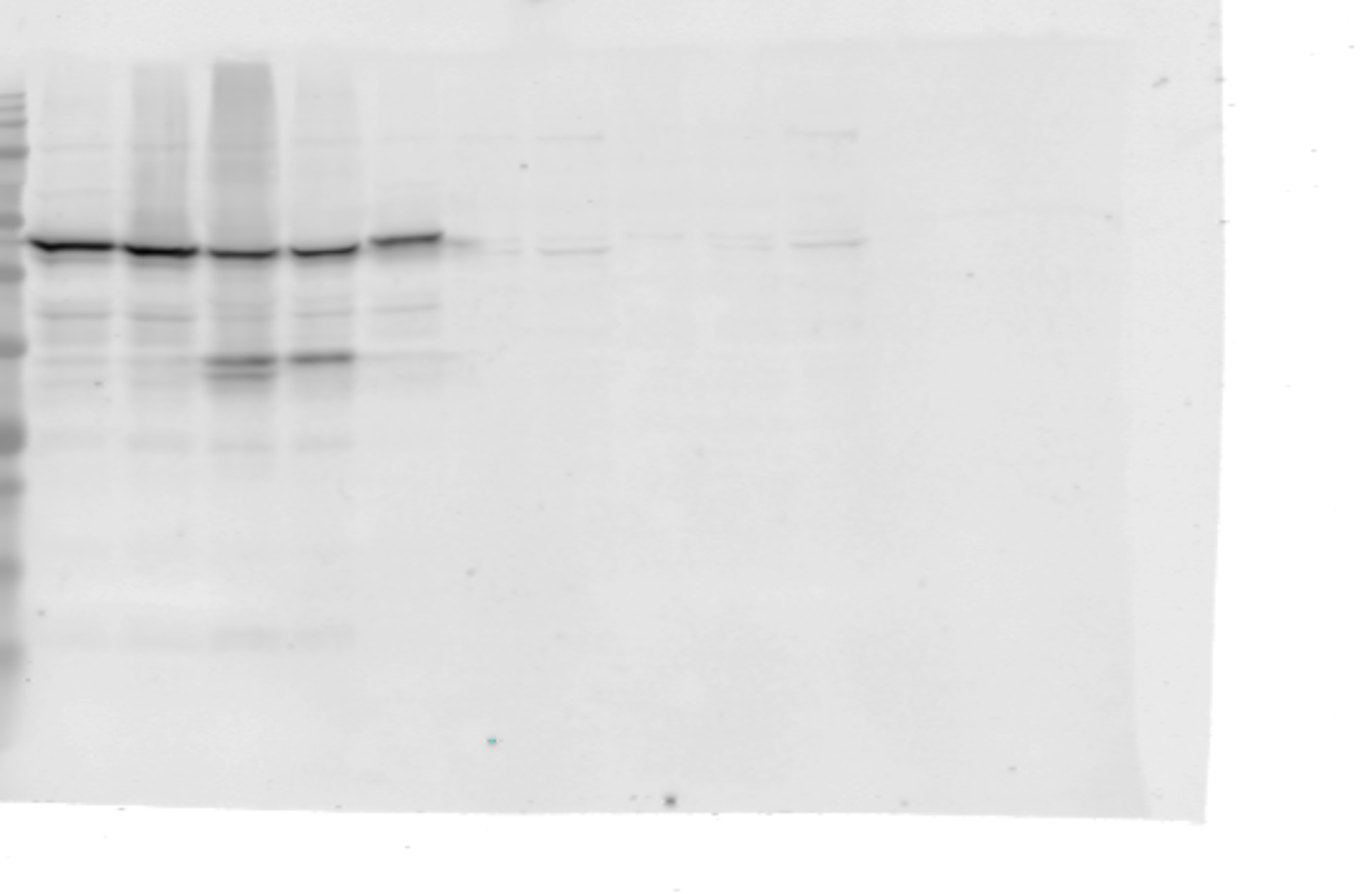

Supplement: Figure 5—source data 2. [file elife-75753-fig5-data2.zip › Source data Figure 5E and G/Fig. 5g_GSDMD.tif]

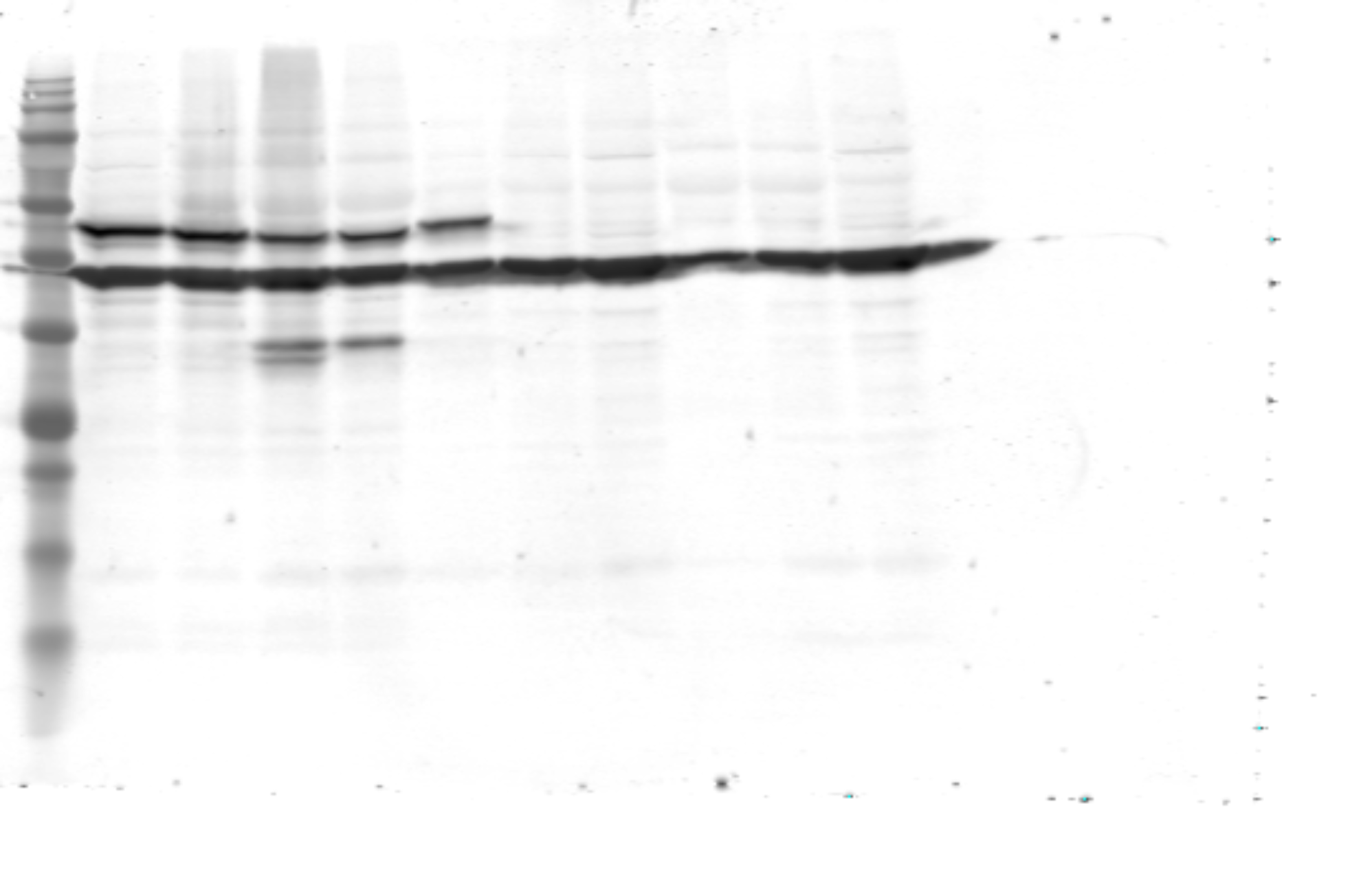

Supplement: Figure 5—source data 2. [file elife-75753-fig5-data2.zip › Source data Figure 5E and G/Fig. 5g_actin.tif]

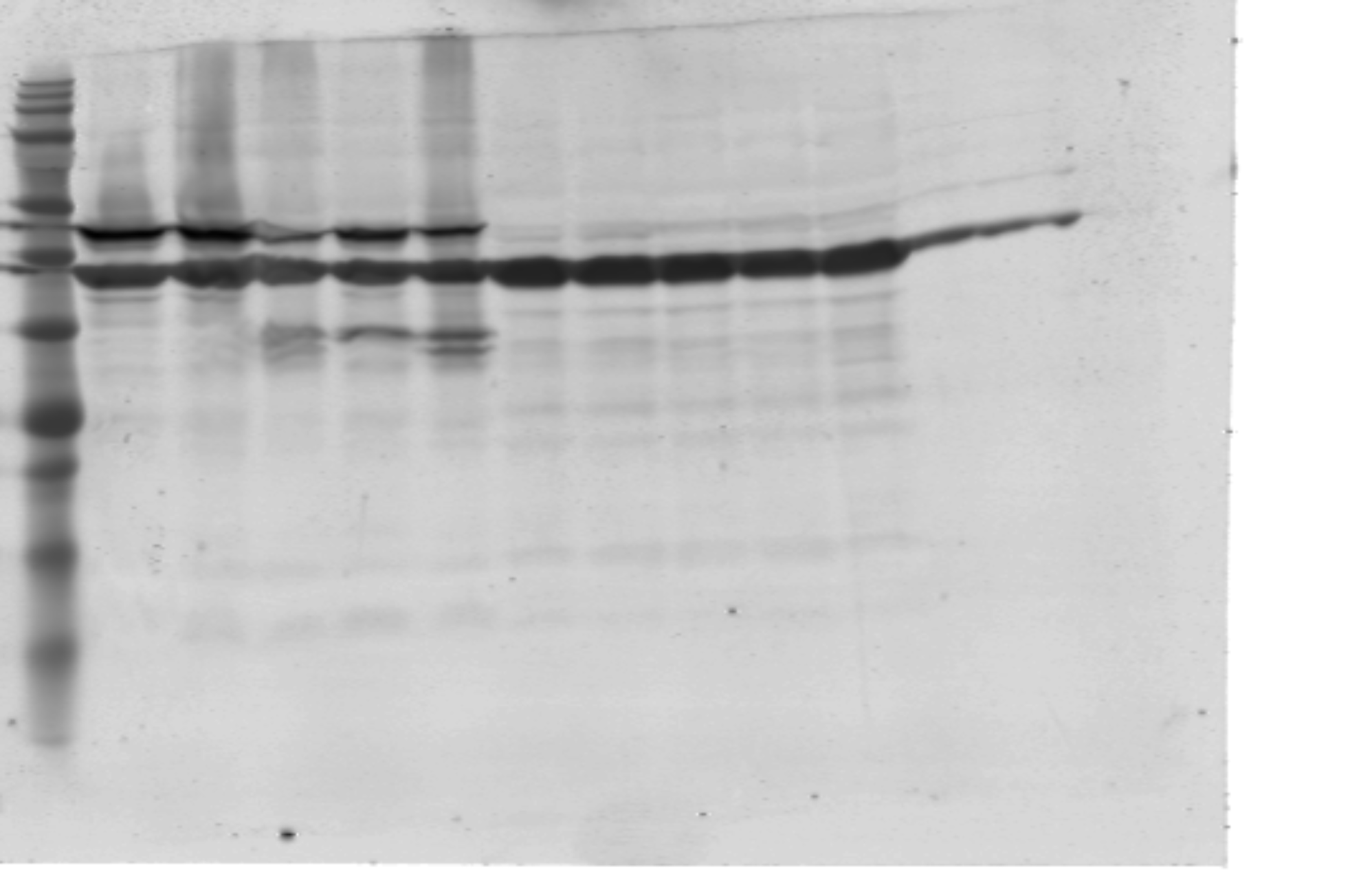

Supplement: Figure 5—source data 2. [file elife-75753-fig5-data2.zip › Source data Figure 5E and G/Fig. 5e_actin.tif]

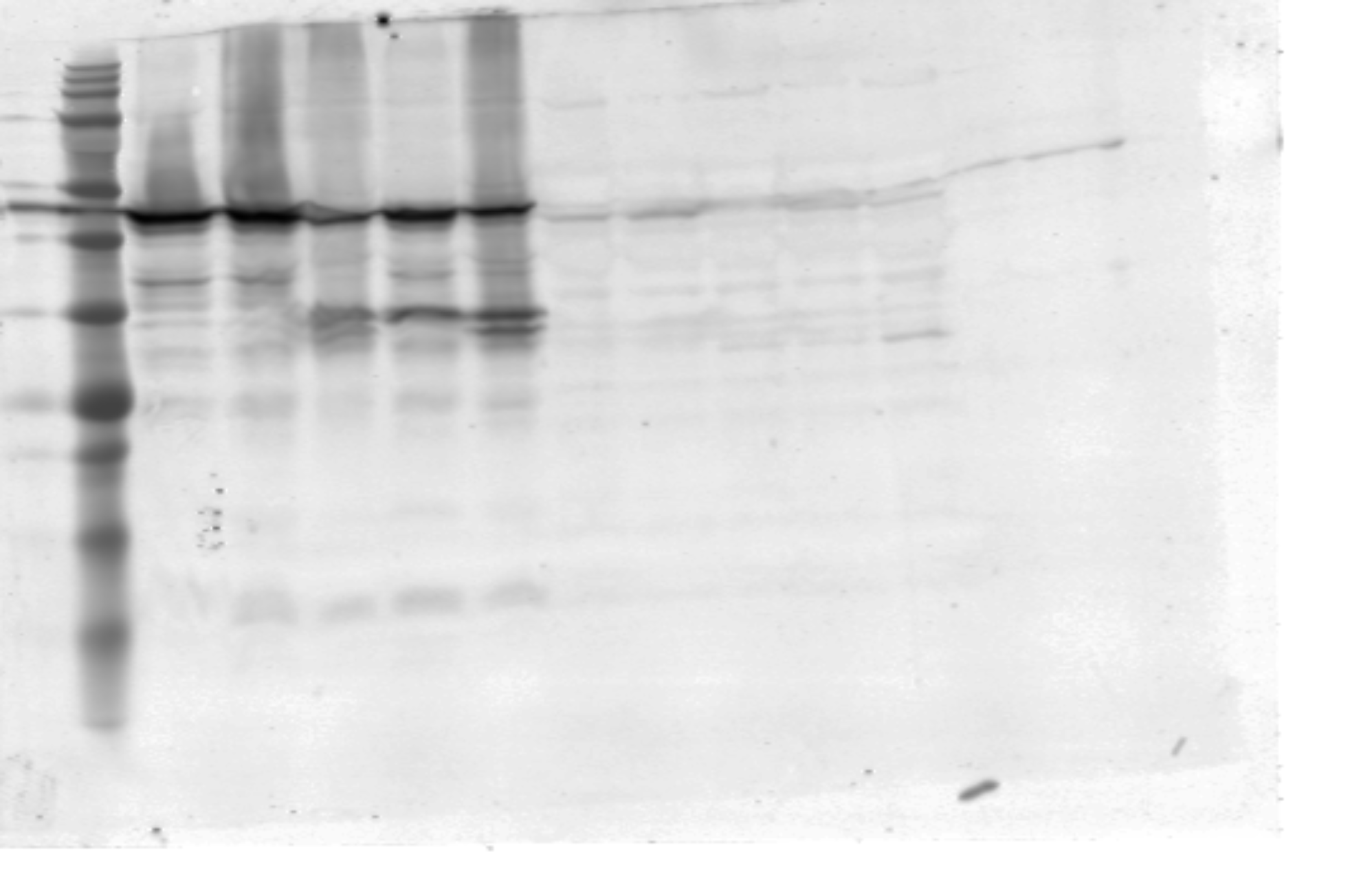

Supplement: Figure 5—source data 2. [file elife-75753-fig5-data2.zip › Source data Figure 5E and G/Fig. 5e_GSDMD.tif]
