# Supplementary material for: Fracture healing is delayed in the absence of gasdermin-interleukin-1 signaling
Source: eLife. 2022 Mar 4;11:e75753. doi: 10.7554/eLife.75753 (PMC8923664; doi:10.7554/eLife.75753)
Supplement: Figure 1—figure supplement 2—source data 2. [file elife-75753-fig1-figsupp2-data2.zip › source data Figure 1-figure supplement 2A and B/Figure S2 WB.pptx]

## Slide 1
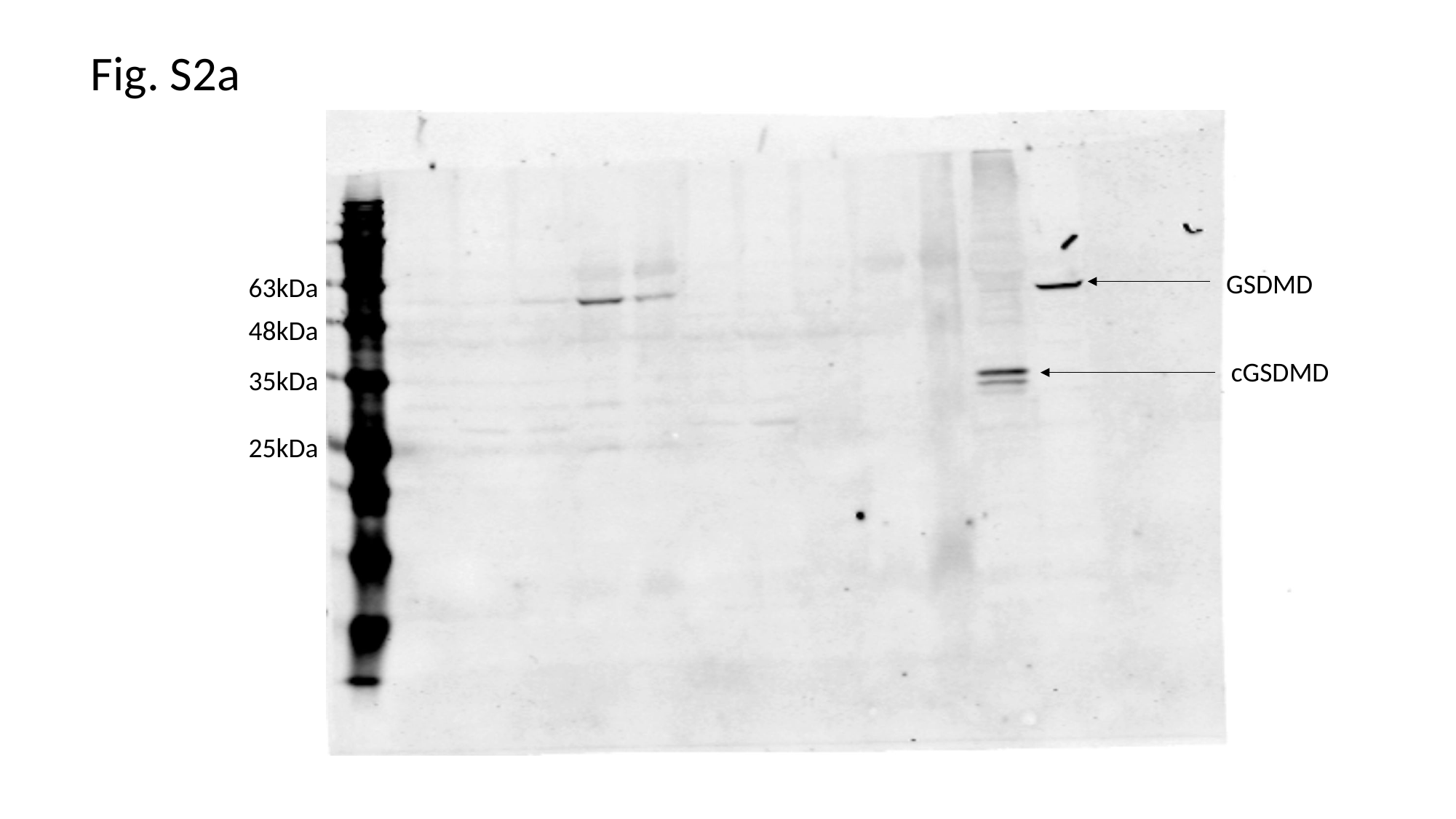

Fig. S2a
GSDMD
63kDa
48kDa
cGSDMD
35kDa
25kDa

## Slide 2
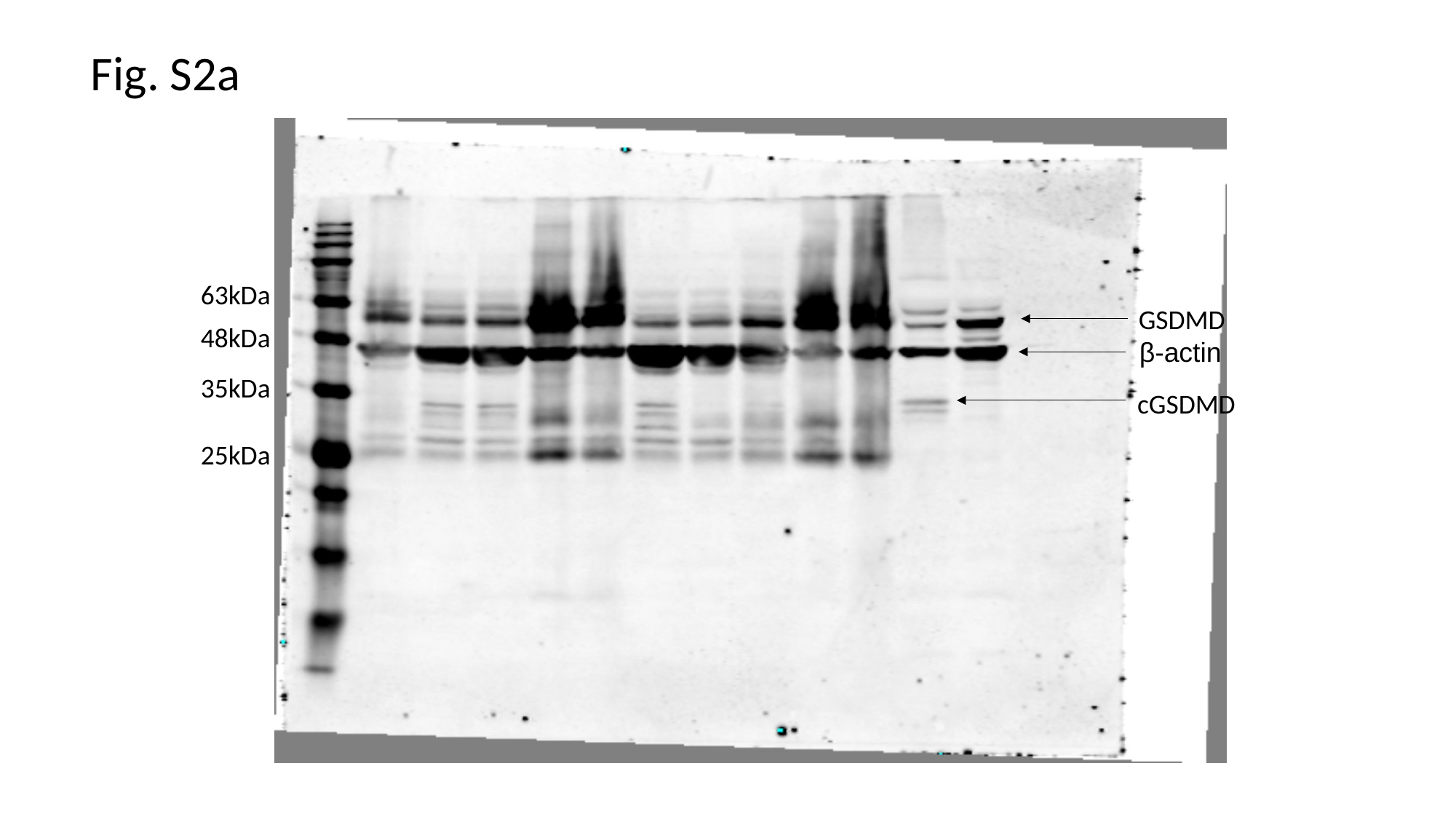

Fig. S2a
63kDa
GSDMD
48kDa
β-actin
35kDa
cGSDMD
25kDa

## Slide 3
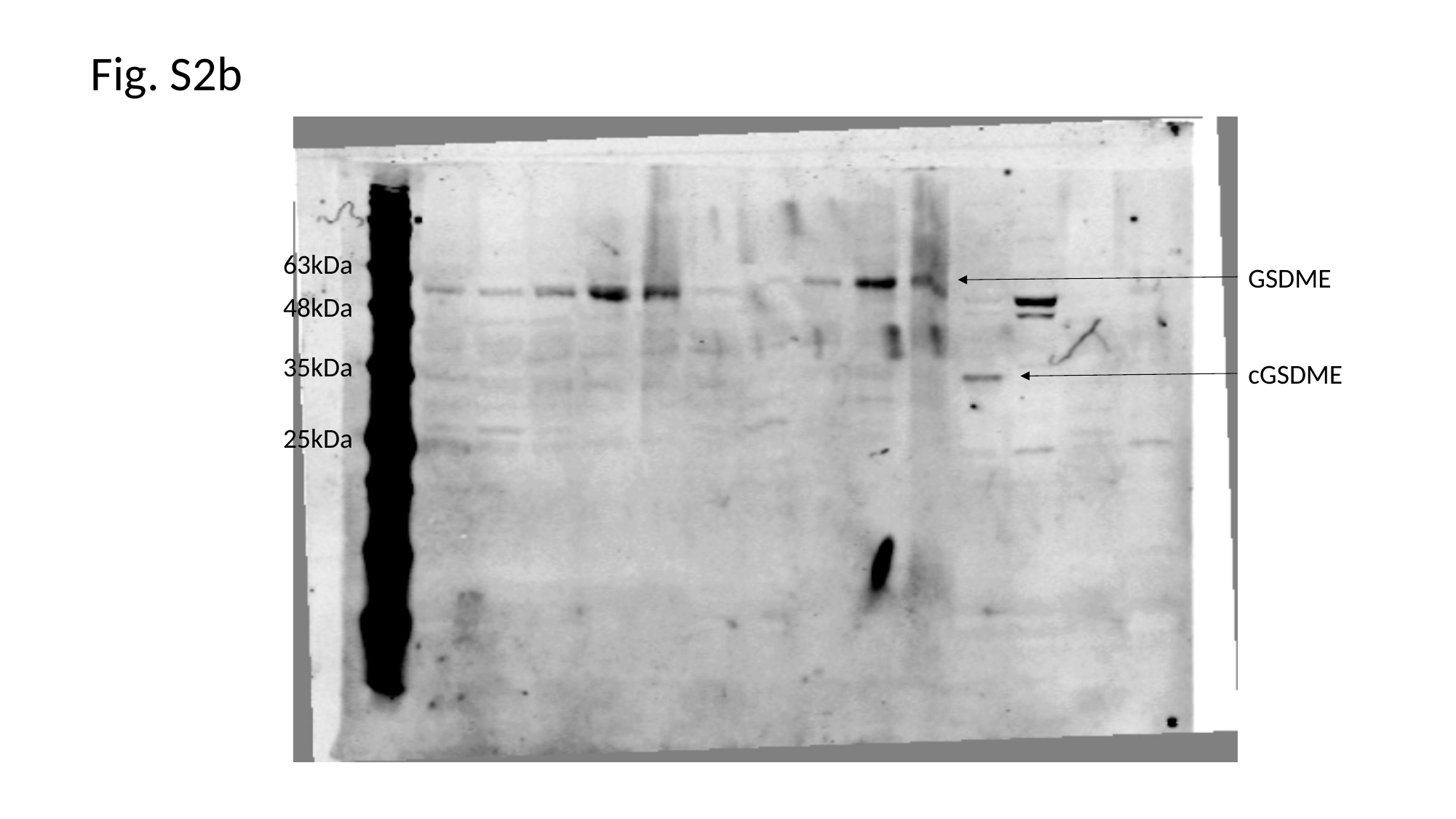

Fig. S2b
63kDa
GSDME
48kDa
35kDa
cGSDME
25kDa

## Slide 4
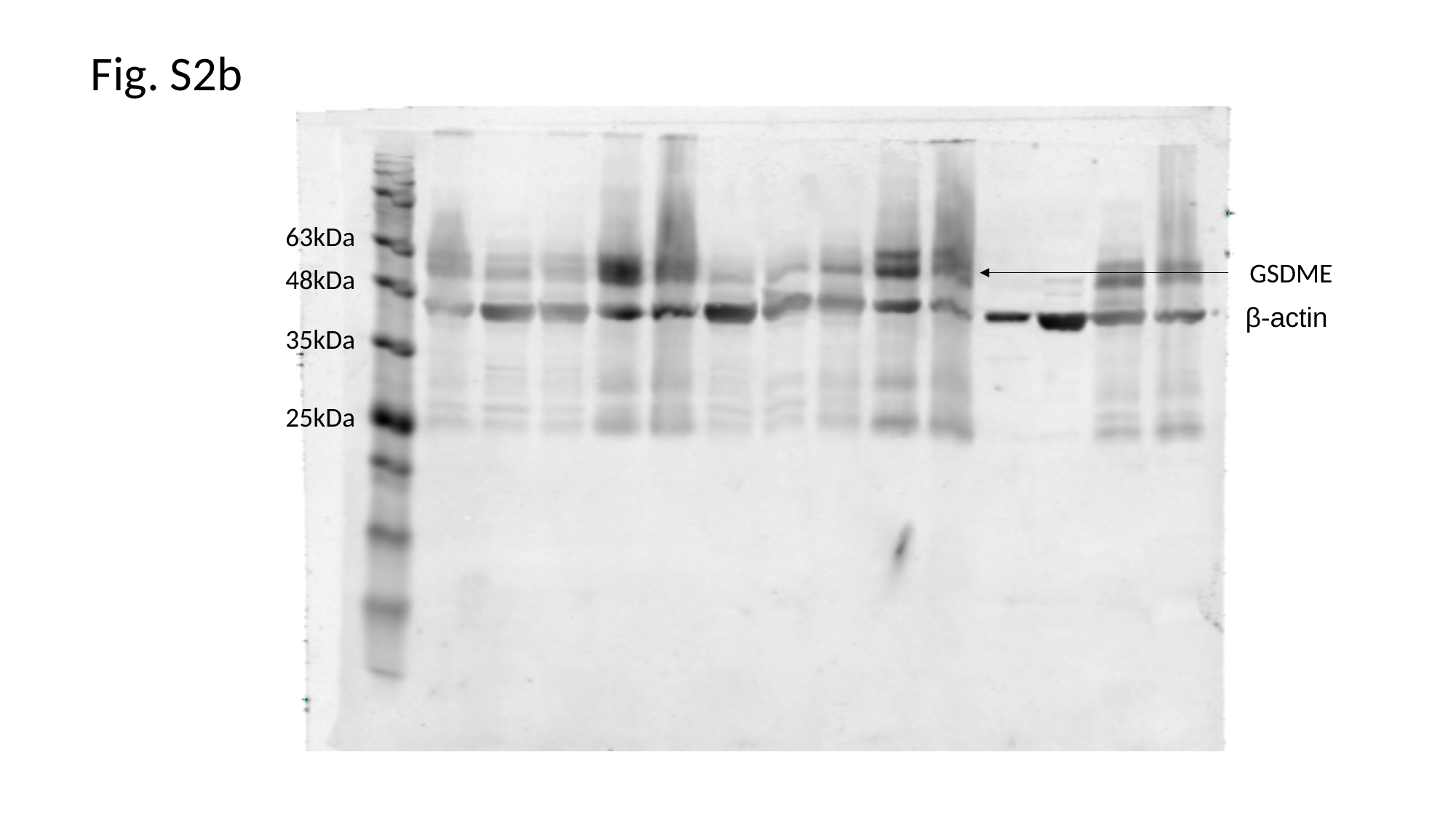

Fig. S2b
63kDa
GSDME
48kDa
β-actin
35kDa
25kDa
