# Supplementary material for: Fracture healing is delayed in the absence of gasdermin-interleukin-1 signaling
Source: eLife. 2022 Mar 4;11:e75753. doi: 10.7554/eLife.75753 (PMC8923664; doi:10.7554/eLife.75753)
Supplement: Figure 5—source data 2. [file elife-75753-fig5-data2.zip › Source data Figure 5E and G/Figure 5 WB.pptx]

## Slide 1
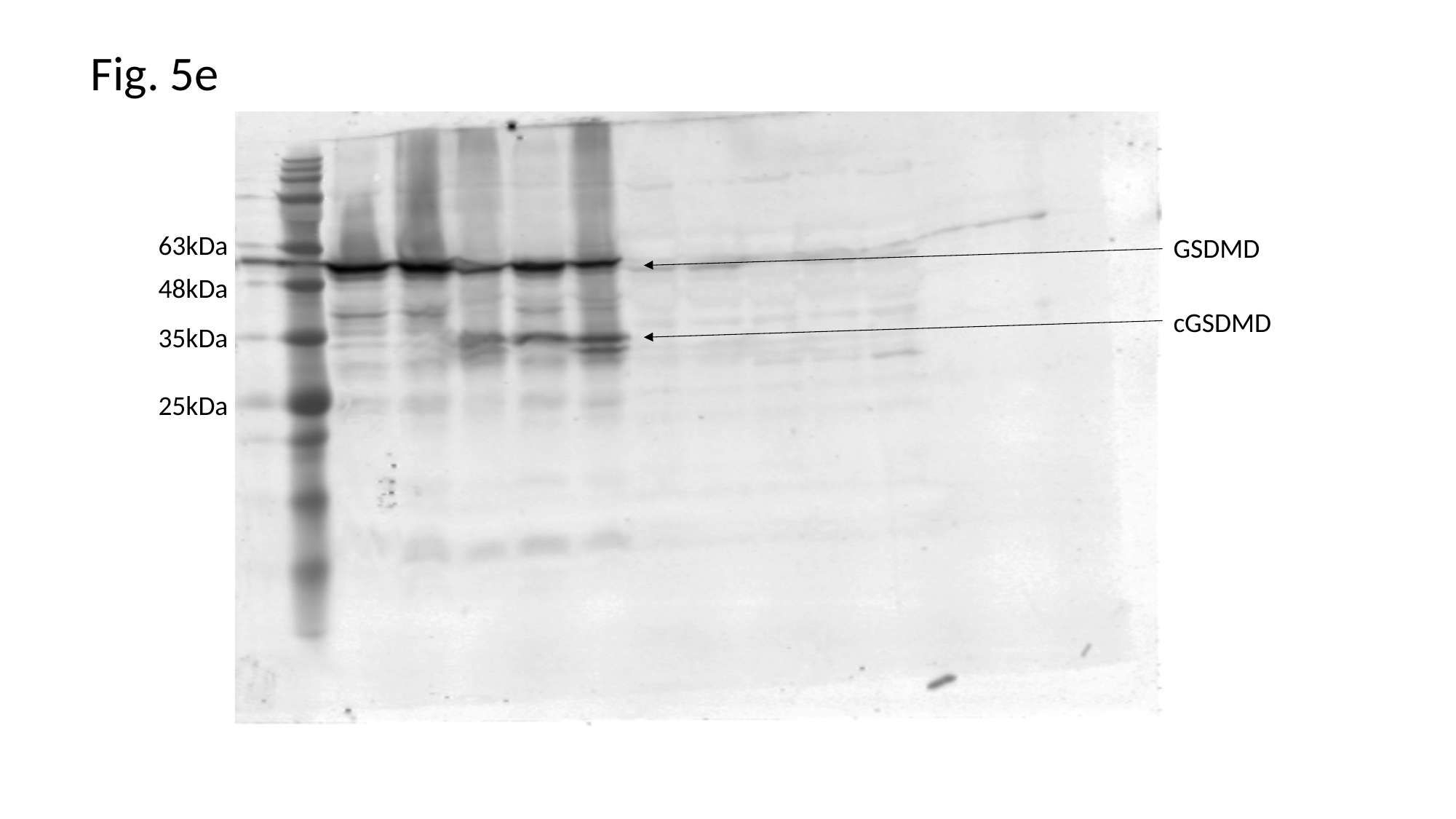

Fig. 5e
63kDa
GSDMD
48kDa
cGSDMD
35kDa
25kDa

## Slide 2
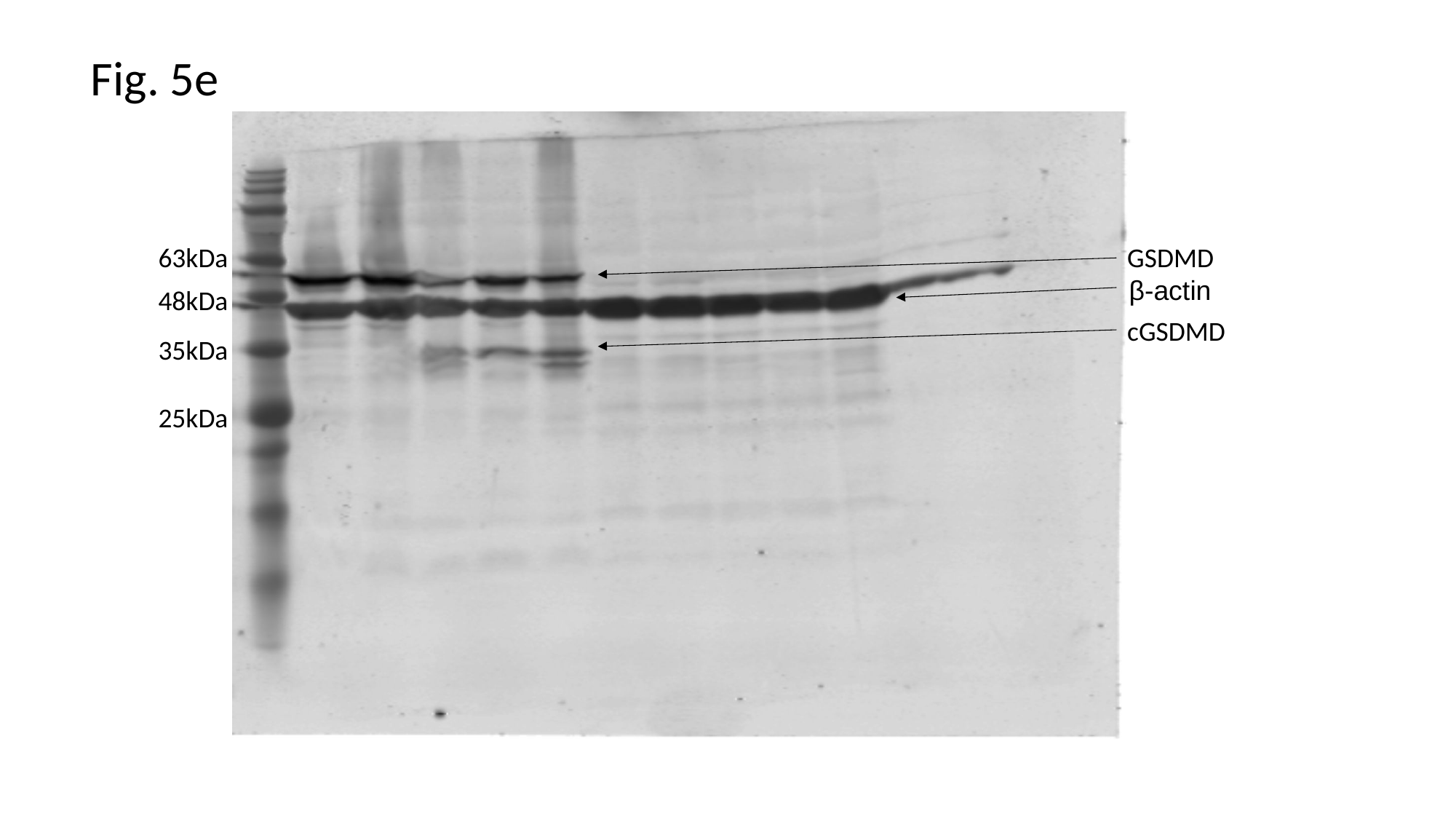

Fig. 5e
63kDa
GSDMD
β-actin
48kDa
cGSDMD
35kDa
25kDa

## Slide 3
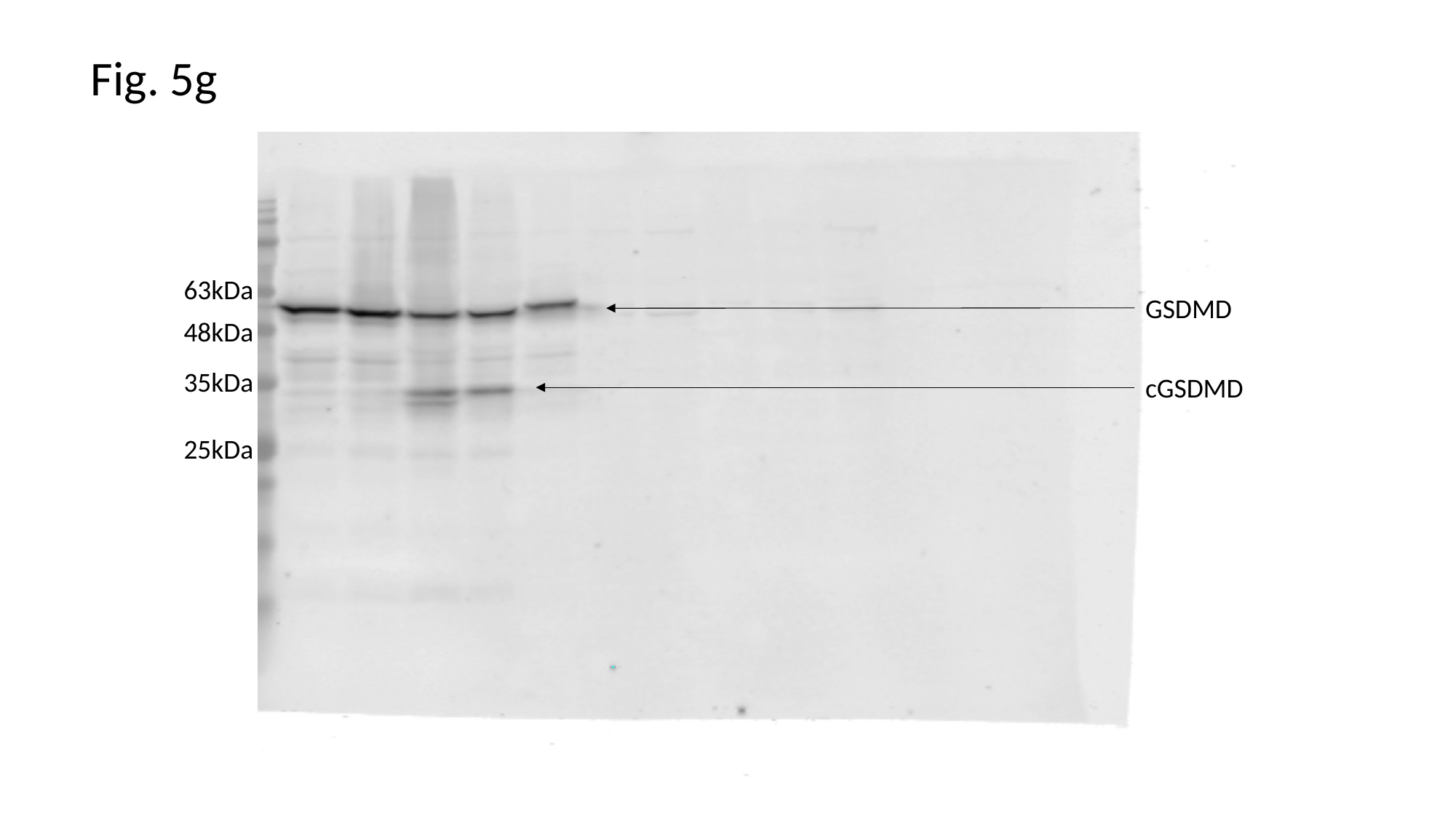

Fig. 5g
63kDa
GSDMD
48kDa
35kDa
cGSDMD
25kDa

## Slide 4
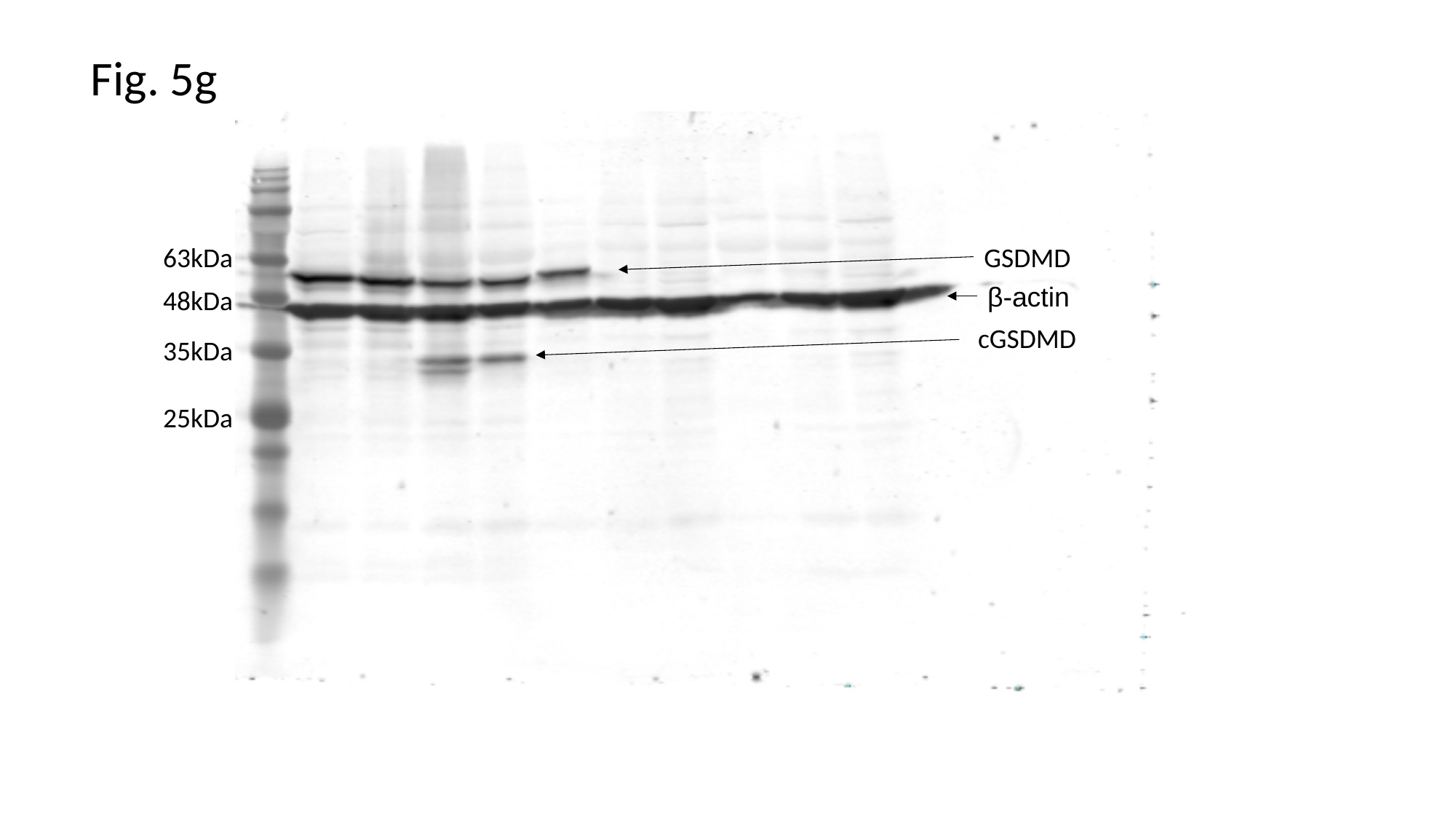

Fig. 5g
63kDa
GSDMD
β-actin
48kDa
cGSDMD
35kDa
25kDa
